# Production of Triple-Gene (GGTA1, B2M and CIITA)-Modified Donor Pigs for Xenotransplantation

**DOI:** 10.3389/fvets.2022.848833

**Published:** 2022-04-28

**Authors:** Kaixiang Xu, Honghao Yu, Shuhan Chen, Yaxuan Zhang, Jianxiong Guo, Chang Yang, Deling Jiao, Tien Dat Nguyen, Heng Zhao, Jiaoxiang Wang, Taiyun Wei, Honghui Li, Baoyu Jia, Muhammad Ameen Jamal, Hong-Ye Zhao, Xingxu Huang, Hong-Jiang Wei

**Affiliations:** ^1^Yunnan Province Key Laboratory for Porcine Gene Editing and Xenotransplantation, Yunnan Agricultural University, Kunming, China; ^2^Yunnan Province Xenotransplantation Research Engineering Center, Yunnan Agricultural University, Kunming, China; ^3^Faculty of Animal Science and Technology, Yunnan Agricultural University, Kunming, China; ^4^College of Biotechnology, Guilin Medical University, Guilin, China; ^5^College of Veterinary Medicine, Yunnan Agricultural University, Kunming, China; ^6^School of Life Science and Technology, ShanghaiTech University, Shanghai, China

**Keywords:** GGTA1, B2M, CIITA, major histocompatibility complex, pig

## Abstract

Activation of human immune T-cells by swine leukocyte antigens class I (SLA-I) and class II (SLA-II) leads to xenograft destruction. Here, we generated the GGTA1, B2M, and CIITA (GBC) triple-gene-modified *Diannan* miniature pigs, analyzed the transcriptome of GBC-modified peripheral blood mononuclear cells (PBMCs) in the pig's spleen, and investigated their effectiveness in anti-immunological rejection. A total of six cloned piglets were successfully generated using somatic cell nuclear transfer, one of them carrying the heterozygous mutations in triple genes and the other five piglets carrying the homozygous mutations in GGTA1 and CIITA genes, but have the heterozygous mutation in the B2M gene. The autopsy of GBC-modified pigs revealed that a lot of spot bleeding in the kidney, severe suppuration and necrosis in the lungs, enlarged peripulmonary lymph nodes, and adhesion between the lungs and chest wall were found. Phenotyping data showed that the mRNA expressions of triple genes and protein expressions of B2M and CIITA genes were still detectable and comparable with wild-type (WT) pigs in multiple tissues, but α1,3-galactosyltransferase was eliminated, SLA-I was significantly decreased, and four subtypes of SLA-II were absent in GBC-modified pigs. In addition, even in swine umbilical vein endothelial cells (SUVEC) induced by recombinant porcine interferon gamma (IFN-γ), the expression of SLA-I in GBC-modified pig was lower than that in WT pigs. Similarly, the expression of SLA-II DR and DQ also cannot be induced by recombinant porcine IFN-γ. Through RNA sequencing (RNA-seq), 150 differentially expressed genes were identified in the PBMCs of the pig's spleen, and most of them were involved in immune- and infection-relevant pathways that include antigen processing and presentation and viral myocarditis, resulting in the pigs with GBC modification being susceptible to pathogenic microorganism. Furthermore, the numbers of human IgM binding to the fibroblast cells of GBC-modified pigs were obviously reduced. The GBC-modified porcine PBMCs triggered the weaker proliferation of human PBMCs than WT PBMCs. These findings indicated that the absence of the expression of α1,3-galactosyltransferase and SLA-II and the downregulation of SLA-I enhanced the ability of immunological tolerance in pig-to-human xenotransplantation.

## Introduction

Worldwide, there is an increasing number of patients with end-stage organ failure in urgent need of transplants, but the number of organs or tissues available from deceased or living human donors is limited (1). Xenotransplantation using genetically modified pig organs or tissues is increasingly close to the clinic. The inactivation of the GGTA1 gene effectively prevents hyperacute rejection (HAR and FDA recently approved that the α-Gal-free pig could potentially be used as a biomedical source) (https://www.fda.gov/news-events/press-announcements/fda-approves-first-its-kind-intentional-genomic-alteration-line-domestic-pigs-both-human-food). Currently, the porcine kidney with α-Gal-free was performed with a short-term *ex vivo* perfusion of a brain-dead patient's blood, indicating no signs of immediate rejection (2). However, other immunological rejection, such as human adaptive immune response, is also important for the long-term survival of xenografts, which can be overcome by the overexpression of other humanized genes and/or deficiency of other porcine endogenous genes. Furthermore, it has been suggested that prolonged survival of xenografts in xenotransplantation needs more genetic modifications under the background of GGTA1 knockout (3, 4).

T-cell-mediated immune responses belong to adaptive immune response and are closely related to the expression of major histocompatibility complex (MHC) genes, including MHC class I, class II, and class III (5, 6). MHC class I and class II proteins share the task of presenting antigens on the cell surface for recognition by CD8^+^ and CD4^+^ T-cells, respectively (7). It has been reported that porcine MHC molecules, including MHC class I and class II, known as swine leukocyte antigen class I and class II (SLA-I and SLA-II), can cross-react with anti-human leukocyte antigen antibodies and trigger human T-cell responses after xenotransplantation (8, 9). The SLA-I is a heterotrimeric complex composed of a heavy α-chain, a light β-chain, and a variable short peptide, expressing on all nucleated cells. The porcine β2-microglobulin (B2M) is a highly conserved gene encoding the β-chain of SLA-I and thus is a potential target for reducing or eliminating the SLA-I expression on the cell surface (10). The SLA-II had high homology with humans and expressed a variety of subtypes on the cell membrane, including SLA-II DRA, DRB1, DQA, and DQB1 (11). Additionally, their expression on spatiotemporal and quantitative aspects is highly governed by the MHC class II transactivator, the CIITA gene (12, 13).

To alleviate the adaptive immune response for xenotransplantation, the pigs carrying B2M-null (14) or expressing human dominant-negative CIITA (hCIITA-DN) (15) have been produced, which significantly diminished the expression of SLA-I or SLA-II. These porcine xenografts can escape from the human T cell-mediated immune response to some extent, resulting in the prolonged survival of xenografts. Therefore, the deletion of GGTA1, B2M, and CIITA genes in pigs could further improve the immune compatibility between humans and pigs more effectively to prolong graft survival.

In this study, to provide an available donor pig for xenotransplantation research, we simultaneously targeted the porcine GGTA1, B2M, and CIITA genes by the CRISPR/Cas9 system and generated the α-Gal, SLA-II-deficient and SLA-I low *Diannan* miniature pigs by somatic cell nuclear transfer (SCNT). We performed RNA sequencing (RNA-seq) of PBMCs in the pig's spleen and analyzed the effects of gene modifications on pig health. We further assessed their effectiveness in anti-immunological rejection by an antibody-antigen-binding assay and mixed lymphocyte reaction (MLR). These results suggested that, although these gene modifications impaired pig health, the pigs were helpful for protecting from human antibody-binding cytotoxicity and cellular immune responses in xenotransplantation.

## Materials and Methods

### Animals and Chemicals

Pigs used in this study were approved by the Animal Care and Use Committee of Yunnan Agricultural University (permission code: YAUACUC01; publication date: 10 July 2013). All chemicals were purchased from Sigma-Aldrich (St. Louis, MO, USA) unless otherwise stated.

### Design of sgRNA Targeting GGTA1, B2M, and CIITA Genes and Construction of Plasmid Vectors

The specific sgRNAs targeting GGTA1, B2M, and CIITA were designed as follows: GGTA1-sgRNA, GCTACAGGCCTGGTGGTACA; B2M-sgRNA, GAAGGTTCAGGTTTACTCAC; and CIITA-sgRNA, TCAACTGCGAACAGTTCAGC. A total of two complementary DNA oligos for generating sgRNAs were annealed. Subsequently, these double-strand DNAs were subcloned into the pGL3-U6-sgRNA (Addgene no.: 51133) vector to generate the reconstructed plasmid vectors. The reconstructed pGL3-U6-GGTA1-sgRNA, pGL3-U6-B2M-sgRNA, and pGL3-U6-CIITA-sgRNA plasmids were confirmed by sequencing. Then, a high concentration (~2,000 ng/μl) of sgRNA and pST1374-NLS-flag-linker-Cas9 (Addgene no.: 44758) plasmids was prepared for cell transfection.

### Cell Culture, Transfection, Selection, and Identification

Pig fetal fibroblasts (PFFs) were prepared as previously described (16). The day before cell transfection, PFFs were thawed and cultured in DMEM supplemented with 10% fetal bovine serum (FBS). Approximately 7 × 10^5^ cells suspended in the electro-transfection buffer containing pGL3-U6-GGTA1-sgRNA (5 μg), pGL3-U6-B2M-sgRNA (5 μg), pGL3-U6-CIITA (5 μg), and pST1374-NLS-flag-linker-Cas9 (5 μg) were electroporated at 250 V for 20 ms with a Gene Pulser Xcell electroporator (Bio-Rad Gene Pulser Xcell, USA).

After electroporation, the cells were plated into a T25 flask for 24 h in DMEM supplemented with 10% FBS. Then, 3 μg/ml Puromycin and 5 μg/ml Blasticidin S were added into the medium for 24−48 h to select successfully transfected cells. Subsequently, the survived cells were digested and about 80 cells were seeded into 100-mm-diameter culture dish for 8 days. At last, the cell colonies were seeded individually into 48-well plates to isolate single-cell colonies. Single-cell-derived colonies were harvested after 3 days of culture, and the colonies were genotyped by PCR, T7 endonuclease I cleavage assay (T7EI), and sequencing. The biallelic GGTA1, B2M, and CIITA knockout (GTKO/B2MKO/CIITAKO) cell colonies were selected as the donor cell for SCNT.

### SCNT and Embryo Transfer

Oocyte collection, *in vitro* maturation, SCNT and embryo transfer were performed as described in our previous studies (17). Briefly, the cultured cumulus oocyte complex (COCs) were freed of cumulus cells by treatment with 0.1% (w/v) hyaluronidase. The first polar body contained in the oocytes and the adjacent cytoplasm was enucleated *via* gentle aspiration using a beveled pipette in TLH-PVA. Donor cells from a GTKO/B2MKO/CIITAKO-positive fibroblast cell line were inserted into the perivitelline space of an enucleated oocyte. The reconstructed embryos were fused with a single direct current pulse of 200 V/mm for 20 μs using the Electro Cell Fusion Generator (LF201, NEPA GENE Co., Ltd., Japan) in fusion medium. Then, the embryos were cultured for 0.5–1 h in PZM-3 and were activated with a single pulse of 150 V/mm for 100 ms in the activation medium. The embryos were equilibrated in PZM-3 supplemented with 5 μg/ml cytochalasin B for 2 h at 38.5°C in a humidified atmosphere with 5% CO_2_, 5% O_2_, and 90% N_2_ (APM-30D, ASTEC, Japan) and then cultured in PZM-3 medium with the same culture conditions described above until embryo transfer. The SCNT embryos were surgically transferred into the oviducts of the recipients.

### Identification of GBC-Modified Piglets

After birth of cloned piglets, their ear tissues were collected, and DNA was extracted using a Tissue DNA Kit (TaKaRa, Japan). The GBC-modified piglets and wild-type cloned piglets were identified by assessing the GGTA1 (Forward primer: 5'-ACAGCAACAGACGTCTCTCATC-3' Reverse primer: 5'-CTTGAAGCACTCCTGAGTGATG-3'), B2M (Forward primer: 5'-GGAAACGAATCCGACTGGTAAC-3' Reverse primer: 5'-GTGGACCAGAAGGTAGAAAGAC-3'), and CIITA (Forward primer: 5'-GATGGACCTGGCTGGAGAAGAAGAG-3' Reverse primer: 5'-CGTGGTTACTCGTCAGGGTTGTTAC-3') genes by RT-PCR, T7EI digestion, and Sanger sequencing.

### Quantitative Polymerase Chain Reaction

The heart, liver, kidney, spleen, and lung tissues were collected from GBC-modified pigs, including pigs P1, P3, P4, and P5 provided in Supplementary Table 1, and WT pigs. Total RNAs were isolated using the TRIzol reagent (Transgen Up, China) according to the manufacturer's instructions. Complementary DNA (cDNA) was synthesized from total RNA using a PrimeScript RT reagent Kit (TaKaRa, Japan) and was used as a template to perform qPCR in SYRB green-based qPCR instrument (CFX-96, Bio-Rad, USA). The reaction was performed in a 20 μl reaction mixtures comprising 10 μl of 2 × SYBR (TaKaRa, Japan), 1 μl of cDNA, 1 μl of forward primer, 1 μl of reverse primer, and 7 μl of ddH_2_O. The reaction program is as follows: 95°C for 30 s, followed by 40 cycles of 95°C for 10 s, and 62°C for 45 s. The relative expression levels of target genes were quantified by 2–ΔΔCt. The primers are listed in Supplementary Table 2.

### CFSE-Based Mixed Lymphocyte Reaction

The human blood was collected from a healthy volunteer. The peripheral blood mononuclear cells (PBMCs) of WT, GBC-modified piglet P4P3 (Supplementary Table 1), and humans were isolated from heparinized blood using the PBMC separation medium kit (Cat# LTS1110, TBD science, China). The PBMCs of WT and GBC-modified pigs, respectively, were treated with 30 μg/ml mitomycin C as stimulator cells. In addition, the human's PBMCs were labeled with 2.5 μm CFSE (Cat# 65-0850-84, Invitrogen) as the responder cells. Then, the 2 × 10^4^ porcine PBMCs per well treated with mitomycin C and 1 × 10^5^ CFSE-labeled human PBMCs were co-cultured in 200 μl RPMI-1640 medium containing 10% FBS at 37°C and in dark conditions. Then, 1.2 × 10^5^ stimulator cells per well were cultured as the negative control and 1.2 × 10^5^ responder cells per well were cultured as the positive control. After 3 days of culture, cells were harvested and performed flow cytometry using a CytoFLEX flow cytometer (Beckman Coulter). The cell proliferation rate was calculated according to the following formula:


$$\begin{array}{l} \text{Cell proliferation rate}=\left(\frac{\text{NPCMC}}{\frac{1}{1.2}\;*\;NPCPC+\frac{0.2}{1.2}\;*\;NPCNC}-1\;\right)\;*\;100\%. \end{array}$$

NPCMC, numbers of progeny cells of mixed culture; NPCPC, numbers of progeny cells of positive control; NPCNC, numbers of progeny cells of the negative control. Progeny cells refer to proliferating cells with a decreased CFSE-labeled fluorescence intensity during cell culture.

### Immunohistochemistry of Tissue Sections

The kidney tissues from GBC-modified pig, P4 (Supplementary Table 1) and WT pig were excised and fixed in 4% paraformaldehyde for 24 h. The tissues were embedded with paraffin and cut into 5 μm. After dewaxing and hydration, sections were incubated in 3% H_2_O_2_ solution for 30 min and washed with phospate-buffered solution (PBS) for three times (each time 3 min). After that, the sections were blocked in PBS containing 5% BSA for 15 min at room temperature. Finally, the tissue sections were incubated with 5 μg/ml anti-gal antibody (ALX-801–090, Abcam, UK) at 4°C overnight. After washing with PBS for three times, sections were incubated with 5 μg/ml HRP-conjugated goat anti-rabbit or -mouse IgG antibody (KIT-9901, Elivision TM plus Polyer HRP IHC Kit, Fuzhou, China) for 20 min. After washing three times again, sections were stained with fresh DAB (KIT-9901, Elivision TM plus Polyer HRP IHC Kit, Fuzhou, China) solution in dark for 5 min. Then, sections were washed with PBS for 3 min for three times, stained with hematoxylin, and imaged by OLYMPUS BX53 fluorescence microscope.

### Immunofluorescence

The paraffin-embedded tissue blocks from GBC-modified pig, P4P3 (Supplementary Table 1), and WT pigs were cut into 5 μm, transferred to glass slides, dewaxed using xylene and gradient alcohol, put into a microwave oven to retrieve antigens with EDTA buffer (pH 8.0; Servicebio Bio, China) at 92–98°C for 15 min, and cooled at room temperature. Then, sections were washed with PBS for three times (each time 3 min), incubated with autofluorescence quencher A (Servicebio Bio, China) at room temperature in dark for 15 min, washed with PBS for three times again, incubated with FBS at room temperature for 30 min, and dried. The dried sections were incubated with the corresponding antibodies.

For detection of SLA-I and SLA-II molecules, FITC-conjugated anti-SLA-I (Cat# MCA2261GA, Bio-rad) and anti-SLA-II DR (Cat# MCA2314GA, Bio-Rad) antibodies were diluted with PBS containing 10% FBS (v/v = 1:200) and used to incubate sections at 4°C in dark for 2 h, and a negative control was incubated with PBS containing 10% FBS.

For detection of B2M and CIITA protein, 1% Triton X-100 was used to punch cell membrane of tissues for 15 min after retrieving antigens. The primary antibodies of anti-B2M (Cat# abs126086a, Absin Bioscience) and anti-CIITA (Cat# sc-13556, Santa Cruz Biotechnology) were diluted with PBS containing 10% FBS (v/v = 1:200) and used to incubate sections at 4°C overnight. Then, sections were washed with PBS for three times, and CY3-conjugated goat anti-rabbit IgG antibodies (Servicebio Bio, China) were diluted with PBS (v/v = 1:200) and used to incubate sections in dark for 2 h.

Then, sections were washed with PBS three times and stained with DAPI (Servicebio Bio, China) for 3 min. After washing with PBS for 1 min, autofluorescence quencher B (Servicebio Bio, China) was added for 5 min and washed three times again. Finally, sections were mounted with antifluorescence quencher (Servicebio Bio, China) and imaged using an OLYMPUS BX53 fluorescence microscope.

### Human Serum-Mediated Antibody-Binding Assay

The mixed human serum (Cat# HSER-P50ML) was purchased from ZenBio, Inc., USA. The serum was inactivated at 56°C for 30 min and was diluted at 1:4 in staining buffer (PBS containing 1% FBS). In addition, porcine fibroblasts derived from the GBC pig, P4P3 (Supplementary Table 1), and WT and human fibroblasts were collected, washed two times, and resuspended in staining buffer. Then, 1 × 10^5^ cells were incubated with 100 μl inactive human serum (test group) or 100 μl PBS (negative control) for 45 min at room temperature. Then, cells were washed with cold staining buffer to terminate reaction and incubated with goat anti-human IgM-FITC (Cat# 2020-02, 1:100 dilution, SouthernBiotech) and goat anti-human IgG Alexa Fluor 647 (Cat# A21249, 1:200 dilution, Invitrogen) for 30 min at 4°C. Finally, cells were washed with cold staining buffer for two times, centrifuged at 400 × g for 4 min, and resuspended with 200 μl PBS for a CytoFLEX flow cytometer (Beckman Coulter, USA).

### Flow Cytometry

All flow cytometry assays were performed by CytoFLEX flow cytometer (Beckman Coulter, USA). For detection of SLA-I, SLA-II DQ, and SLA-II DR surface molecules on porcine umbilical vein endothelial cells derived from the GBC pig, P4P3 (Supplementary Table 1) and WT, cells were cultured in the cell medium (Cat# TM002, abmGood) containing 10% FBS (Cat# VS500T, Ausbian) and 1% Pen-Strep solution (Cat# 03-031-1BCS, Biological Industries) with or without 100 ng/ml of recombinant pig interferon-gamma (IFN-γ; Cat# PPP022, Bio-rad) for 3 days. After collection, 1 × 10^5^ cells were incubated with mouse anti-pig SLA class I (Cat# MCA2261GA, 1:100, Bio-Rad), mouse anti-pig SLA class II DQ (Cat# MCA1335GA, 1:100, Bio-rad), and mouse anti-pig SLA class II DR (Cat# MCA2314GA, 1:100, Bio-rad) for 1 h at 4°C, respectively. After that, cells were washed two times with PBS and centrifuged at 1,400 rpm for 5 min. Then, cells were labeled with the secondary antibody FITC goat anti-mouse IgG (Cat# AS001, ABclonal) for 1 h at room temperature.

For detection of CD4 and CD8 T-cell subtypes in PBMCs derived from the blood of WT and GBC-modified pigs by flow cytometry, PBMCs were isolated from whole blood with the porcine PBMC separation medium kit (Cat# LTS1110, TBD science, China). Then, 2 × 10^5^ cells per sample were incubated with anti-CD3 antibody conjugated with FITC (Cat# 559582, 1:40, BD Pharmingen), anti-CD4 antibody conjugated with PE-Cy7 (Cat# 561473, 1:40, BD Pharmingen), and anti-CD8 antibody conjugated with APC (Cat# 561475, 1:40, BD Pharmingen) individually and in combination in dark for 2 h at 4°C. All data acquired from flow cytometry were analyzed using FlowJo VX software.

### Transcriptome Profiling of PBMC in Pig's Spleen

The three GBC-modified piglets, namely, GBC_1, GBC_2, and GBC_3, were generated by recloning from the piglet named P4P1 (Supplementary Table 1) and sacrificed for sample collection at 6, 32, and 34 days after birth. In addition, three WT piglets, namely, WT_1, WT_2, and WT_3, were sacrificed at 6, 19, and 34 days after birth. The PBMCs of the pig's spleen were isolated and extracted using the PBMC separation medium kit (Cat# LTS1110P, TBD science, China) according to the manufacturer's instructions. Total RNAs were extracted using TRIzol reagent (Invitrogen, USA) following the manufacturer's procedure. The total RNA quantity and purity were quantified using NanoDrop ND-1000 (NanoDrop, USA). The RNA integrity was assessed by Bioanalyzer 2100 (Agilent, USA) and confirmed by electrophoresis with denaturing agarose gel. The high-quality RNA samples with RIN number >7.0 were used to construct the sequencing library. Poly (A) RNA was purified from 1 μg total RNA using Dynabeads Oligo (dT) 25-61005 (Thermo Fisher, USA) using two rounds of purification. Then, the poly(A) RNA was fragmented into small pieces using Magnesium RNA Fragmentation Module (NEB, Cat# e6150, USA) under 94°C for 5–7 min. Then, the cleaved RNA fragments were reverse-transcribed to create the cDNA by SuperScript™ II Reverse Transcriptase (Invitrogen, Cat# 1896649, USA), which were next used to synthesize U-labeled second-stranded DNAs with *Escherichia coli* DNA polymerase I (NEB, Cat# m0209, USA), RNase H (NEB, Cat# m0297, USA), and dUTP solution (Thermo Fisher, Cat# R0133, USA). An A-base was then added to the blunt ends of each strand, preparing them for ligation to the indexed adapters. Each adapter contains a T-base overhang for ligating the adapter to the A-tailed fragmented DNA. Single- or dual-index adapters are ligated to the fragments, and size selection was performed with AMPureXP beads. After the heat-labile UDG enzyme (NEB, Cat# m0280, USA) treatment of the U-labeled second-stranded DNAs, the ligated products were amplified with PCR by the following conditions: initial denaturation at 95°C for 3 min; eight cycles of denaturation at 98°C for 15 s, annealing at 60°C for 15 s, and extension at 72°C for 30 s; and then final extension at 72°C for 5 min. The average insert size for the final cDNA library was 300 ± 50 bp. At last, we performed the 2 × 150 bp paired-end sequencing (PE150) on an Illumina Novaseq™ 6000 (LC-Bio Technology CO., Ltd., Hangzhou, China) following the vendor's recommended protocol.

Fastp software (http://opengene.org/fastp/fastp) was used to obtain the clean reads by removing the reads that contained adaptor contamination, low-quality bases, and undetermined bases with default parameters. Then, clean reads were mapped using the Sus scrofa genome 11.1 as a reference with HISAT2 (https://ccb.jhu.edu/software/hisat2). The fragments per kilobase of transcript per million fragments mapped (FPKM) value of each gene was calculated using StringTie (https://ccb.jhu.edu/software/stringtie) with default parameters. The differentially expressed genes (DEGs) were selected with fold change >2 or fold change <0.5 and with parametric *F*-test comparing nested linear models (*P*-value < 0.05) by R package edgeR (https://bioconductor.org/packages/release/bioc/html/edgeR.html). The DEGs were annotated by Gene Ontology (GO) functional enrichment and Kyoto Encyclopedia of Genes and Genomes (KEGG) pathway analysis using the topGO package (http://www.geneontology.org) and the online website tool at https://www.kegg.jp/, respectively.

### Statistical Analysis

All data were expressed as mean ± standard error (SE). Independent sample unpaired *t*-test was performed with SPSS 22.0 software package (IBM Crop, Armonk, NY) for the data of qPCR, relative median fluorescence intensity (MFI), and cell proliferation rate. Statistical significance was defined as ^*^P < 0.05, ^**^, ## *P* < 0.01.

## Results

### Generation and Identification of GTKO/B2MKO/CIITAKO Donor Cells and Piglets

**Figure 1:**
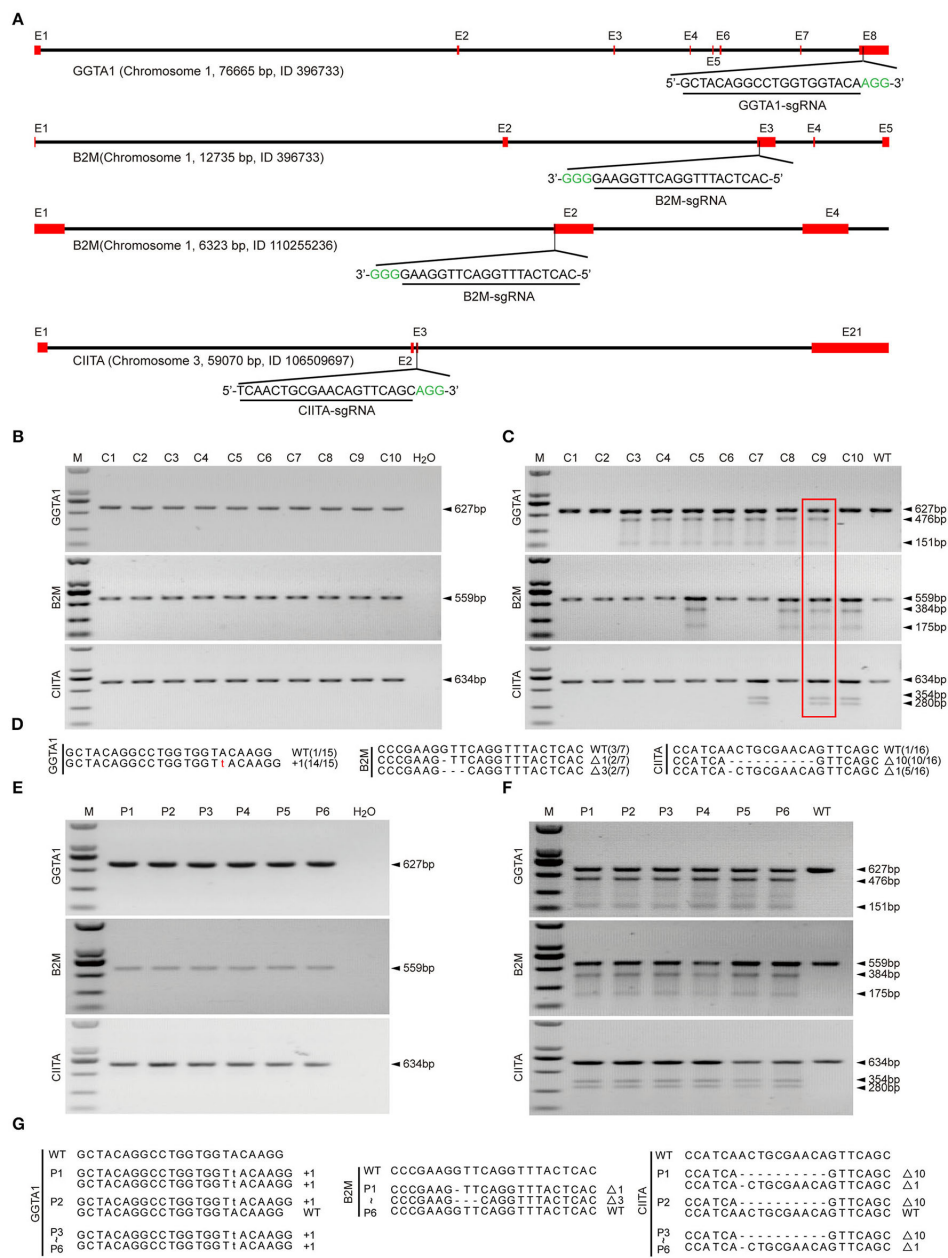
Establishment of GBC-modified cell colonies and generation of GBC-modified pigs. **(A)** Schematic diagram of targeting to porcine GGTA1, B2M, and CIITA genes by the CRISPR/Cas9 system. **(B)** PCR products harboring the targeting regions of GGTA1, B2M, and CIITA genes from cell colonies (M, DNA maker DL2000; C, colony). **(C)** Identification of postive cell colonies by T7EI cleavage assay. The colony C9 is positive for triple-gene modification. **(D)** Genotypes of colony C9 by Sanger sequencing. **(E)** PCR products harboring the targeting regions of GGTA1, B2M, and CIITA genes from cloned pigs (P, pig). **(F)** Identification of cloned pigs by T7EI cleavage assay. **(G)** Genotypes of cloned pigs by Sanger sequencing.

We designed the sgRNAs targeting the GGTA1, B2M, and CIITA genes (Figure 1A). Then, these sgRNA plasmids and Cas9 plasmids were co-transfected into PFFs, and a total of 10 single-cell colonies were obtained after puromycin and Blasticidin S selection. The PCR and T7EI cleavage showed that the colony C9 was confirmed as GTKO/B2MKO/CIITAKO modification (Figures 1B,C). In addition, Sanger sequencing further indicated that the genotypes of GGTA1, B2M, and CIITA at the target sites were WT/+1 bp, WT/−1 bp/−3 bp, and WT/−1 bp/−10 bp in the colony C9, respectively (Figure 1D). Therefore, the cell colony C9 was used as the donor cells for SCNT. The reconstructed embryos were transferred into 13 recipient gilts, five of whom became pregnant, and three delivered, yielding six piglets (P1–P6; Table 1). All these piglets were confirmed by PCR and T7EI cleavage of the target site as the GTKO/B2MKO/CIITAKO-modified individuals (Figures 1E,F). In addition, the Sanger sequencing analysis indicated that the genotypes of the GGTA1 gene were +1 bp/+1 bp in all piglets except in piglet P2 (WT/+1 bp), of the B2M gene were WT/−1 bp/−3 bp in all piglets, and of the CIITA gene were −1 bp/−10 bp in all piglets except in piglet P2 (WT/−10 bp) (Figure 1G). Considering that the B2M gene was not completely knocked out, thereafter, we named our GTKO/B2MKO/CIITAKO pigs as GBC-modified pigs.

**Table 1:** The embryo transfer and generation of GBC-modified piglets.

**Recipients**	**Donor cells**	**No. of transferred embryos**	**Pregnancy (%)**	**Days of pregnancy**	**No. of offspring (alive)**
1	Colony C9	210	–	–	–
2		205	+	119	1(1)
3		300	+	117	1 (Stillborn)
4		280	–	–	–
5		300	–	–	–
6		270	+	129	2(1)
7		270	+	118	1 (Stillborn)
8		280	–	–	–
9		280	–	–	–
10		223	–	–	–
11		300	–	–	–
12		241	+	114	1(1)
13		220	–	–	–
Total		3,379	5(38.5%)		6(3)

### Autopsy of GBC-Modified Pigs

**Figure 2:**
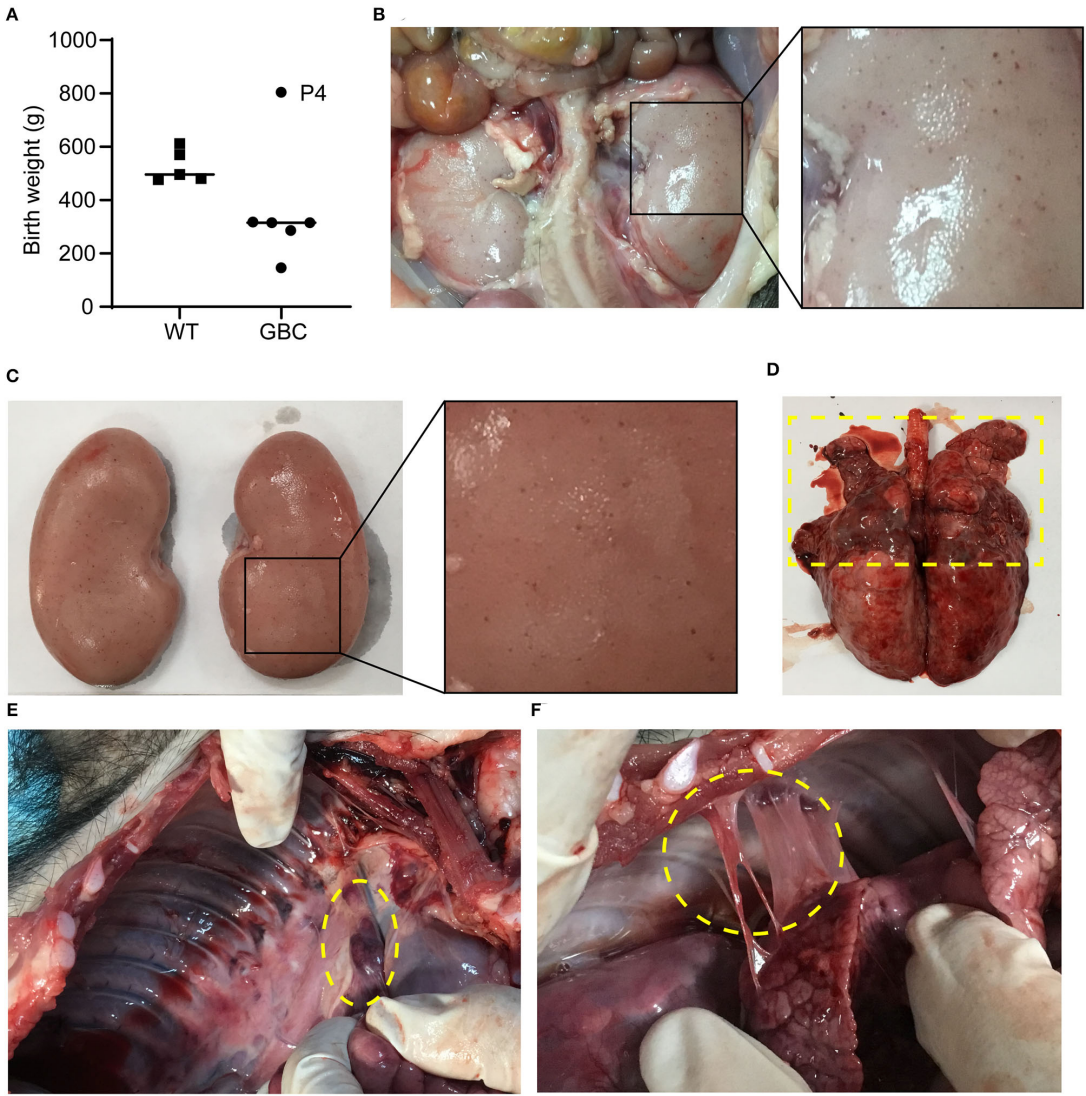
Autopsy of GBC-modified pigs. **(A)** Birth weight of GBC-modified piglets. **(B,C)** A lot of spot bleeding in the kidney of GBC-modified pig P4P2 (13 days). **(D)** The lungs of GBC-modified pig P4P1 (93 days), indicating severe suppuration and necrosis in the upper part. **(E)** Enlarged peripulmonary lymph nodes in pig P4P1. **(F)** Adhesion between the lungs and the chest wall in pig P4P1.

We observed that the birth weight of GBC-modified piglets except for piglet P4 was lower than WT piglets, and the birth weight of the smallest piglets only was 147 g (Figure 2A). The piglet P4 survived for 19 days but other piglets died soon after birth. Next, we recloned the GBC-modified pigs using the fibroblast cells of piglet P4 for donor cells, obtained three live piglets, namely, P4P1, P4P2, and P4P3, and they survived for 93, 13, and 12 days, respectively. We performed the autopsy of piglets P4P1 and P4P2 and found a lot of spot bleeding in the kidney of the piglet P4P2 (Figures 2B,C) and severe suppuration and necrosis in the upper part of the lungs (Figure 2D), enlarged the peripulmonary lymph nodes (Figure 2E) and adhesion between the lungs and chest wall (Figure 2F) in the piglet P4P1, and suspected severe respiratory pathogen infection. These issues suggested that the modification of B2M and CIITA genes affected these procine's health and made them susceptible to pathogenic microorganisms.

### Functional Identification of GGTA1, B2M, and CIITA Genes in GBC-Modified Pigs

**Figure 3:**
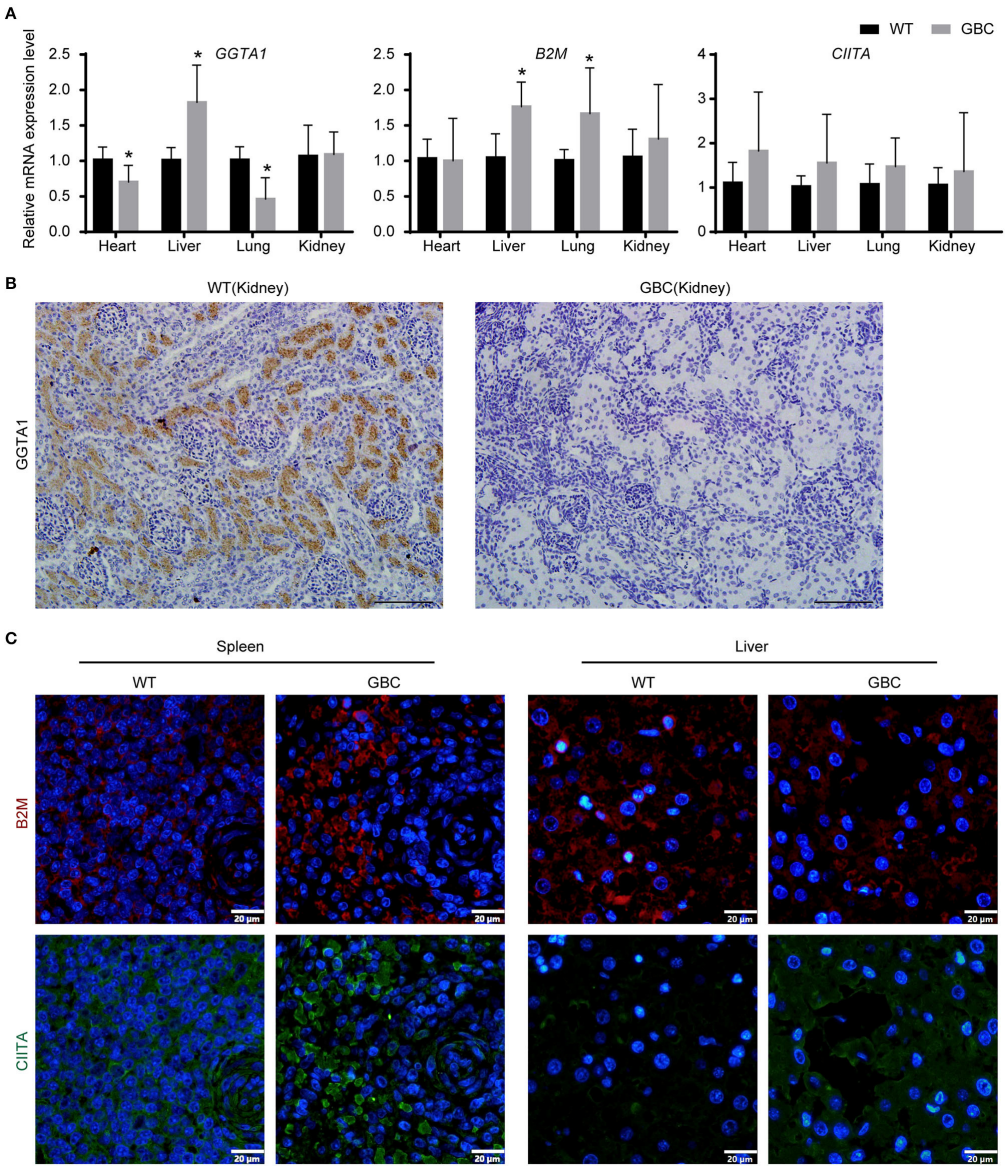
Expression of GGTA1, B2M, and CIITA genes in GBC-modified pigs. **(A)** The mRNA expression levels of GGTA1, B2M, and CIITA genes in the heart, the liver, the lungs, and the kidney (*P < 0.05 vs. WT). **(B)** Immunohistochemical staining of αGal in the kidney (scale bar = 100 μm). **(C)** Immunofluorometric analysis of B2M and CIITA proteins in the spleen and the liver (scale bar = 20 μm).

To verify that the three genes are functionally inactive or impaired in GBC-modified pigs, we first detected their mRNA expression level in tissues. As a result, GGTA1 mRNA expression level was significantly decreased in the heart and the lungs, but that was greatly increased in the liver and was comparable in the kidney when compared to the wild type. Meanwhile, the mRNA expression levels of the B2M and CIITA genes were either elevated or comparable in GBC-modified pigs to WT pigs (Figure 3A). These results indicated that the mutated three genes still can be transcribed into mRNA.

**Figure 4:**
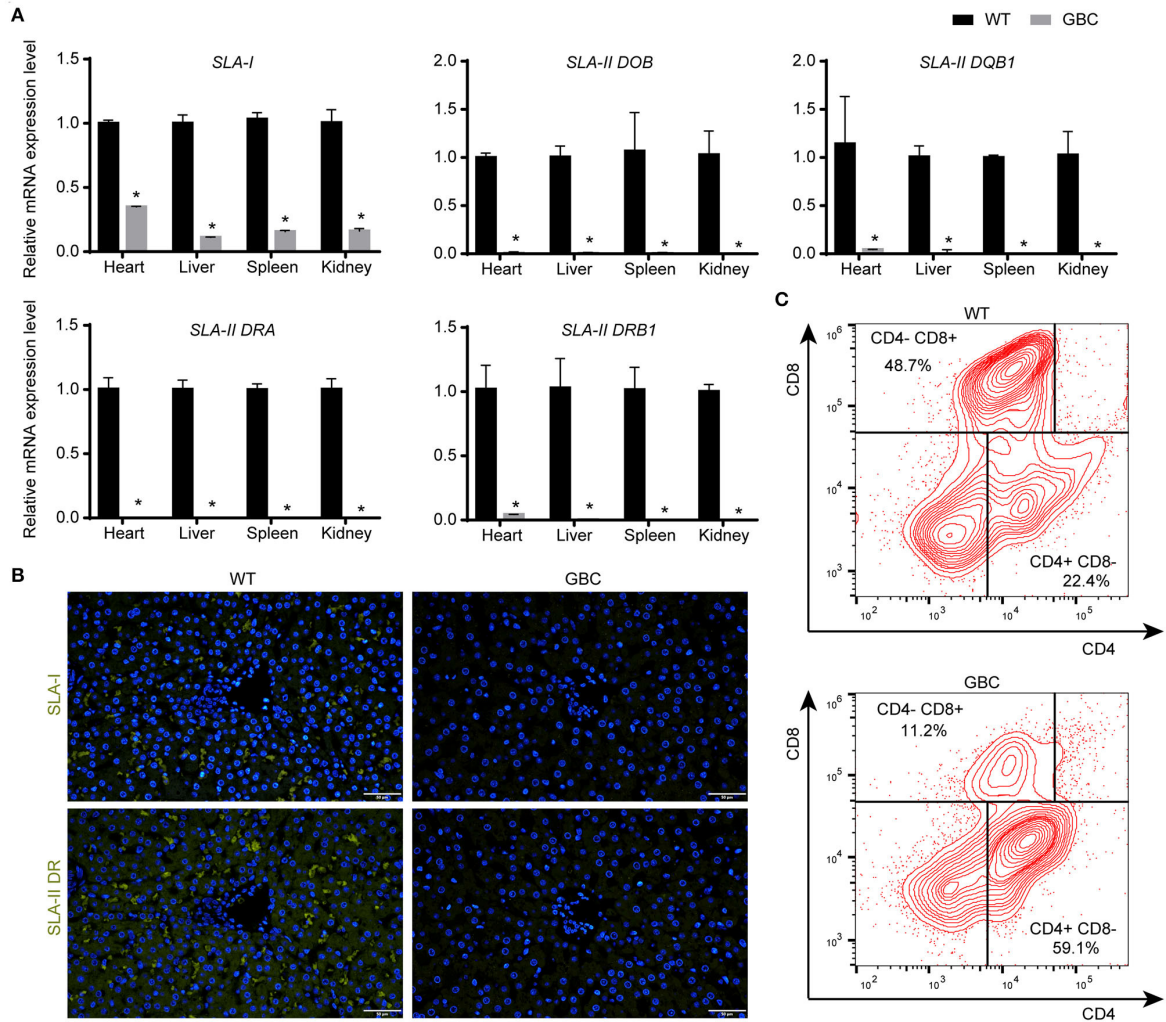
Expression of SLA-I and SLA-II genes, and alteration of T-cell subtype (CD4 and CD8) in GBC-modified pigs. **(A)** The mRNA expression levels of SLA-I and four isoforms of the SLA-II gene in the heart, liver, lung, and kidney (*P < 0.05 vs. WT). **(B)** Immunofluorometric analysis of SLA-I and SLA-II DR molecules in the liver (scale bar = 50 μm). **(C)** The percentage of CD4^+^/CD8^−^ and CD4^−^/CD8^+^ T-cells in PBMCs derived from WT and GBC-modified pigs.

**Figure 5:**
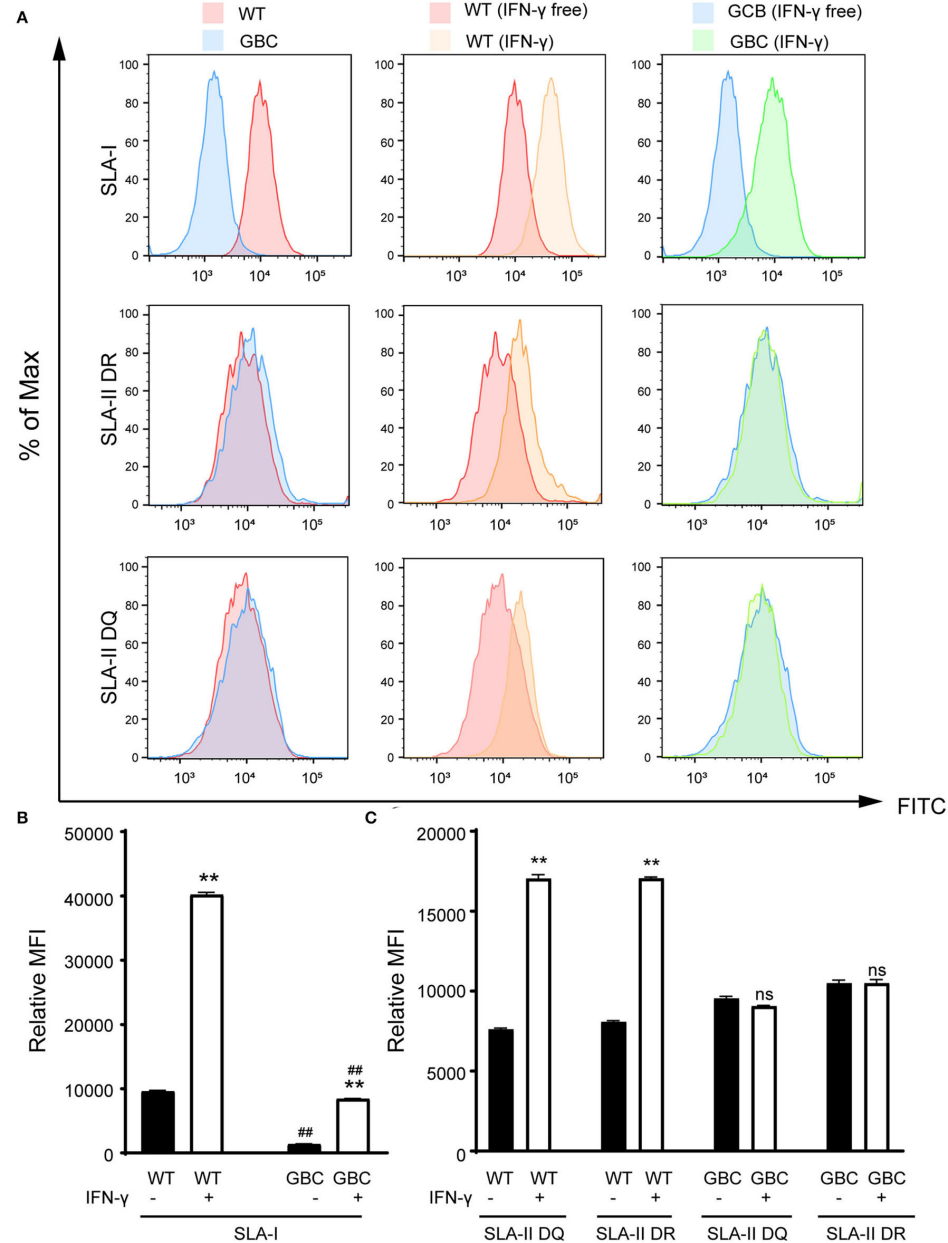
Flow cytometry analysis of expression of SLA-I, SLA-II DR, and SLA-II DQ molecules in the umbilical vein endothelial cells (UVECs) of GBC-modified pigs. **(A)** The expression of the SLA-I molecule is lower in GBC-modified pigs than WT pigs. Additionally, interferon-γ treatment caused an increase in the number of SLA-I molecules in both GBC-modified and WT pig UVECs, but WT had a higher increase than the other. The expression of SLA-II DR and DQ molecules was comparable between GBC-modified and WT pig UVECs. After interferon-γ treatment, the expression of SLA-II DR and DQ molecules was increased in WT pig UVECs but not changed in GBC-modified pig UVECs. **(B,C)** Relative mean immunofluorescence intensity (MFI) of SLA-I **(B)**, SLA-II DR, and SLA-II DQ **(C)** in WT and GBC-modified pig UVECs treated with or without IFN-γ. Data of three dependent experiments are presented. **P < 0.01 vs. IFN-γ-free; ^*##*^*P* < 0.01 vs. WT.

Given that the mRNA cannot reflect whether the mutated B2M and CIITA genes were knocked out, we further detect the protein expression levels of the three genes by immunohistochemistry or immunofluorescence on a tissue level. As a result, the GGTA1 gene was dysfunctional (Figure 3B), but B2M and CIITA proteins still were obviously detected in GBC-modified pigs (Figure 3C). Therefore, we speculated that the B2M and CIITA genes with short-fragment insertion or deletion will accelerate the upregulation of nonsense mRNA and protein expression levels. Given the above results, we directly analyzed the mRNA expression levels of SLA-I and four isoforms of SLA-II (SLA-II DOB, SLA-II DQB1, SLA-II DRA, and SLA-II DRB1) in GBC-modified pigs. The result showed that the heart, liver, spleen, and kidney of GBC-modified pigs had decreased expression levels of SLA-I and SLA-II mRNA than those of WT pigs (Figure 4A). Consistently, the protein expression levels of SLA-I and SLA-II DR in the liver were significantly decreased compared with WT (Figure 4B). We also detected the percentage of CD4 and CD8 T-cells from CD3-positive PBMCs derived from WT and GBC-modified pigs. The result showed that the percentage of CD8-positive cells was decreased compared with WT pigs, demonstrating that the mutation of B2M and CIITA genes led to the impaired CD8 T-cell development in GBC-modified pigs (Figure 4C). Moreover, the SLA-I had a significantly lower level in umbilical vein endothelial cells (UVECs) of GBC-modified pigs than those of WT pigs. After treatment with IFN-γ, SLA-I levels were significantly upregulated in both WT and GBC UVECs, but the higher upregulated SLA-I level of WT UVECs than that of GBC UVECs (Figures 5A,B), suggesting that B2M in CBG pigs was not completely deleted. Although the SLA-II DR and SLA-II DQ had a similar level between WT and GBC UVECs when untreated with IFN-γ, their levels were significantly upregulated in WT UVECs but not in GBC UVECs when treated with IFN-γ (Figures 5A,C), suggesting that the CIITA gene cannot effectively transactivate the expression of SLA-II molecules in GBC UVECs, even with IFN-γ treatment. Therefore, these results finally determined that B2M and CIITA genes in our GBC-modified pigs were dysfunctional or functionally impaired.

### Transcriptome Analysis of PBMC of the Spleen in GBC-Modified Pigs

**Figure 6:**
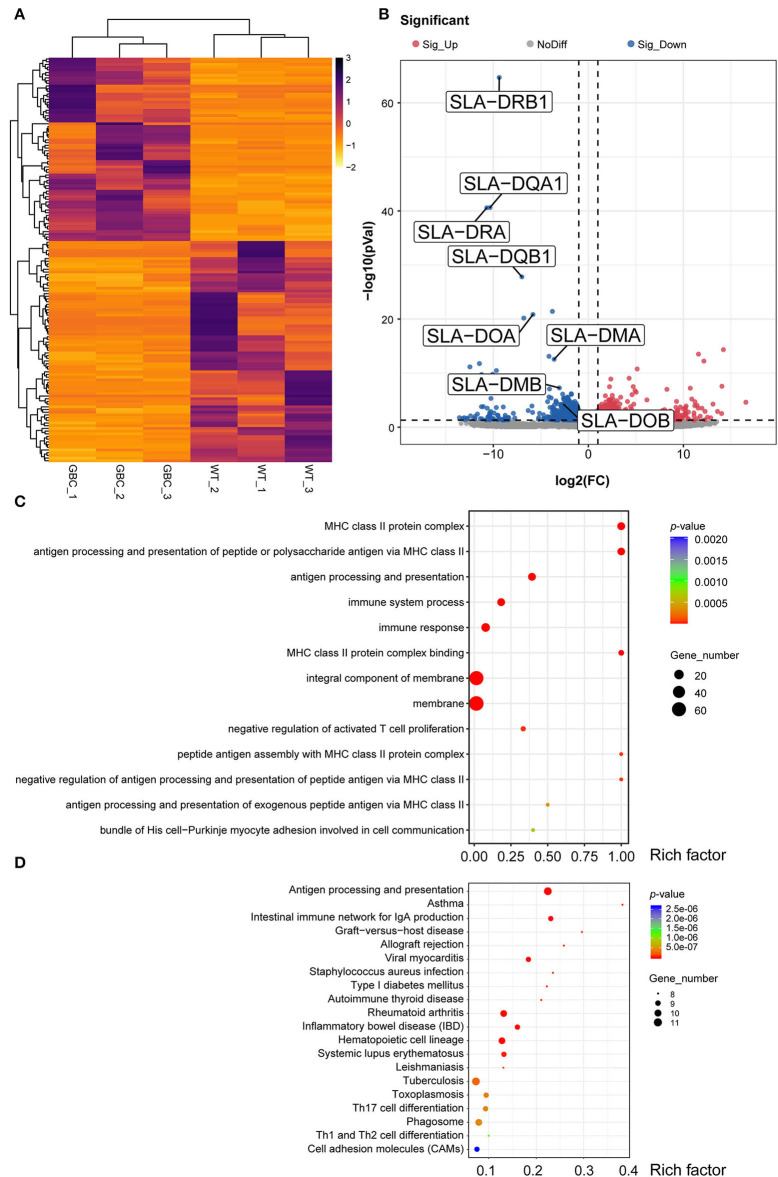
Transcriptome profiling of PBMCs in GBC-modified pig's spleen. **(A)** Heatmap of 150 significant DEGs (*P* < 0.05) between GBC-modified and WT pigs. **(B)** A volcano plot between GBC modified and WT pigs, including 68 upregulated genes and 82 downregulated genes. **(C)** The top 13 Gene Ontology (GO) enrichments of DEGs. The *y*-axis is the name of each category, the *x*-axis is their rich factor. Rich factor = numbers of genes at a category/total numbers of genes at GO analysis. **(D)** The top 20 Kyoto Encyclopedia of Genes and Genomes (KEGG) pathways of DEGs. The *y*-axis is the name of each category, and the *x*-axis is their rich factor. Rich factor = numbers of genes at a pathway/total numbers of genes at KEGG analysis. The number of genes enriched in each category was shown at the size of each circular.

To further investigate the effects of GBC modification on the porcine immune system, we performed RNA-seq and analyzed the transcriptional profile of the spleen PBMC of GBC-modified pigs. The high Pearson correlation coefficient (Supplementary Figure 1A) and no significant differences in the gene expression among the biological replicates (Supplementary Figure 1B) showed that the RNA-seq data were reliable and met the conditions for differential expression analysis. Using the *P*-value (*P* < 0.05) and fold change (FC > 2) as the different standards for analysis, a total of 150 differential expression genes (DEGs) were detected between WT and GBC-modified pig's spleen PBMCs, including 68 upregulated genes and 82 downregulated genes (Figures 6A,B). Notably, a cluster of SLA genes was significantly downregulated in GBC-modified pigs, including SLA-DRB1, SLA-DQA1, SLA-DRA, SLA-DQB1, SLA-DOA, SLA-DMA, SLA-DMB, and SLA-DOB. Additionally, the expression of GGTA1, B2M, and CIITA genes in GBC-modified pig's spleen PBMCs also had no significant change compared with WT (Supplementary Figure 2). These findings were consistent with our qPCR results. Furthermore, the GO analysis of 150 DEGs revealed enrichment of 365 GO terms (*P* < 0.05). The top 20 significant enrichments indicated that CIITA knockout caused a severely impaired immune system, especially in antigen processing and presentation (Figure 6C). The pathway analysis based on the KEGG database revealed that many immune-related and infection-related pathways were enriched (Figure 6D), also suggesting that insufficiency of B2M and CIITA genes may make pigs susceptible to pathogenic microorganisms.

### Immune Response of GBC-Modified Porcine Cells to Mixed Human Serum and Human PBMC

**Figure 7:**
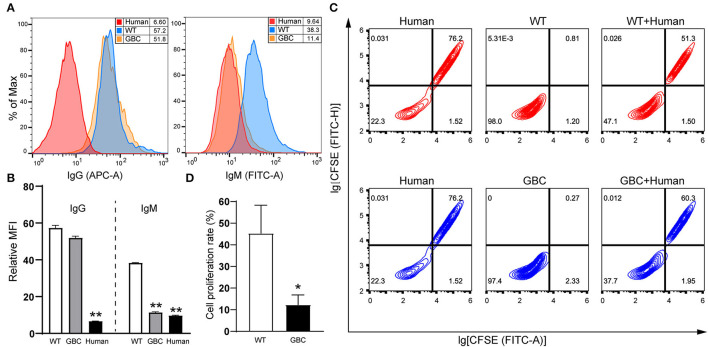
Evaluation of anti-immunological rejection effectiveness of GBC-modified pigs. **(A)** Reduction of human IgM binding to GBC-modified pig fibroblast cells. **(B)** Relative mean immunofluorescence intensity (MFI) of IgG and IgM antibodies binding to WT, GBC-modified, and human fibroblast cells treated with mixed human serum. Data of three dependent experiments are presented (**P < 0.01 vs. WT). **(C)** Proliferation analysis of human peripheral blood mononuclear cells (PBMCs) after stimulation with porcine PBMCs by flow cytometry, and human PBMCs were labeled with CFSE. **(D)** Quantification of the proliferation rate of human PBMCs stimulated with porcine PBMCs (*P < 0.05 vs. WT).

To investigate whether GBC-modified pigs had a weakened xenotransplantation rejection, we performed the IgG and IgM antibody-binding assays of mixed human serum and the mixed lymphocyte reaction between pigs and humans. As a result, the human antibodies IgM but not IgG binding to GBC-modified porcine fibroblasts were obviously decreased compared with WT (Figures 7A,B). After the co-culture of porcine and human PBMCs, the proliferation rate of the human PBMC mixed with GBC porcine PBMCs as the stimulators was significantly lower than that of the human PBMC mixed with WT porcine PBMCs (Figures 7C,D). Therefore, our GBC-modified pigs can attenuate xenogeneic immune rejection.

## Discussion

The development of CRISPR/Cas9 systems makes porcine genome engineering to be simple, accompanied with the increasingly multiple genetically modified pigs available for xenotransplantation research, and xenotransplantation is more likely to be close to the clinic. Here, we generated the GGTA1, B2M, and CIITA triple-gene-modified *Diannan* miniature pigs with CRISPR/Cas9 system in combination with SCNT. Recently, Fu et al. (18) also obtained the pigs with the knockout of GGTA1, B2M, and CIITA genes using the same technology. Differently, they applied the multiple sgRNAs to target each gene, which cloud increase the editing efficiency but simultaneously increased the risk of off-targets. Peng et al. (19) deemed it necessary to minimize the number of sgRNAs for reducing the negative effects caused by the off-target editing when a pool of sgRNAs was used simultaneously. In this study, we only selected one sgRNA for each target gene, aiming to decrease the risk of off-targets. It has been known that there were at least two copies of the B2M gene in the pig genome. For the B2M gene, we designed the single sgRNA for targeting its two copies. Unfortunately, only one copy of the B2M gene was mutated in our GBC-modified pigs, resulting in the reduction of SLA-I expression rather than deficiency. In addition, GGTA1, B2M, and CIITA genes appeared two genotypes of WT and 1-bp insertion (+1/WT), three genotypes of WT, 1- and 3-bp deletion (Δ1/Δ3/WT), and three genotypes of WT, 1- and 10-bp deletion (Δ1/Δ10/WT) in the single-cell colony C9, respectively. Correspondingly, our GBC-modified pigs also showed two genotypes, the pig P2 carrying the genotypes of +1/WT, Δ1/Δ3/WT, and Δ10/WT and the others carrying the genotypes of +1/+1, Δ1/Δ3/WT, and Δ1/Δ10 in GGTA1, B2M, and CIITA genes, respectively. Therefore, we speculated that the colony C9 did not grow from a single cell but rather formed from a mixed cell population in the cell culture.

In the GBC-modified pigs, we found that the mRNA expression levels of GGTA1, B2M, and CIITA genes in some tissues were comparable or upregulated compared with WT pigs. Recently, a similar phenomenon was also reported in zebrafish and mice, in which the short insertions or deletions in some genes triggered genetic compensation that pretends that these mutant mRNAs have no function, thus leading them to transcribe more mRNAs for genetic compensation (20). However, the α1,3-galactosyltransferase was undetectable, and thus, we speculated that the mRNA of the mutated GGTA1 gene was degraded by a nonsense-mediated mRNA decay pathway (21). In addition, the fluorescence intensity of B2M and CIITA was comparable with WT. It might be either due to alternative splicing that produced an isoform protein in the mRNA maturation process or due to translation that might be reinitiated at exons after the modified site to form a truncated protein after mRNA maturation (22, 23). Nevertheless, these isoforms or truncated proteins were dysfunctional, resulting from the reduced SLA-I and SLA-II molecules in GBC-modified pigs. In Figure 5, we found that the expression levels of SLA-II DR and DQ in the GBC UVECs without IFN-γ induction were similar to WT. After treatment with IFN-γ, the expression levels of SLA-II DR and DQ in WT UVECs were upregulated, but no changes were observed in GBC UVECs. The SLA-II molecules were mainly expressed in primarily antigen-presenting cells such as macrophages, dendritic cells, and B cells and often do not express in endothelial cells but can be induced by IFN-γ (24). Based on our results, we suspect that IFN-γ hardly induces SLA-II expression in UVECs porcine, thus leading to minor changes between WT and GBC UVECs. Importantly, the GBC modification can alleviate the immune response to human serum antibodies and PBMCs and contributed to the survival of porcine skin in pig-to-non-human primate xenotransplantation (data not shown). Therefore, we believe that the skin of GBC-modified pigs is valuable for patients with severe burns, especially for patients that are highly sensitized to HLA-I and II.

In pig-to-human xenotransplantation, porcine xenoantigens, including αGal epitopes, can initiate the binding of human natural antibodies, IgG and IgM, thus leading to complement activation and cause HAR. It has been demonstrated that the knockout of the GGTA1 gene can alleviate effectively HAR (25). Here, we also confirmed that the inactivation of the GGTA1 gene can attenuate HAR because the ratio of human IgM binding to fibroblast cells of the GBC-modified pigs was decreased (Figure 7A). In our previous study, simultaneous knockout of GGTA1, CMAH, and β4GalNT2 genes reduced the binding ability of human IgG and IgM by 90% (26), which essentially overcome HAR. Therefore, we suggested that multiple genetically modified donor pigs for xenotransplantation should be considered to delete the two other xenoantigens Neu5Gc and SDa, except αGal epitopes.

The SLA molecules play an important role in cellular immune response in pig-to-human xenotransplantation. To ensure the long-term survival of xenografts, the SLA molecules could be considered for knockout or knockdown. The donor pigs carrying B2M null or SLA-I deficiency had been generated and demonstrated that these genetic modifications can effectively improve the survival of xenografts (14, 27). However, several studies indicated that the inactivation of SLA-I molecules impaired the porcine immune system and led them to susceptibility to infection. In addition, these pigs need to be kept under strict hygienic housing conditions to maintain their healthy growth (22, 28). Our results of the autopsy and transcriptome profiling also demonstrated that B2M and CIITA knockout destroyed the porcine immune system, resulting in susceptibility to pathogenic microorganisms. It has been reported that the survival times of the pigs with SLA-I inactivation did not exceed 4 weeks (28). In this study, the longest survival of a GBC-modified pig (P4P1) with the decreased SLA-I expression was up to 93 days and it grew up to 6.8 kg when reaching sexual maturity. Therefore, we suggested that knockdown rather than knockout of B2M may be more conducive to the survival and breeding process of donor pigs. Of course, we may also consider the overexpression of human HLA molecules instead of deleting SLA molecules to weaken the response of human immune cells (29). On the other hand, the inactivation of CIITA abolished the expression of SLA-II molecules, seriously impaired the differentiation and maturation of porcine CD4 lymphocytes, and was likely to cause pigs to suffer from the bare lymphocyte syndrome (30). Therefore, it seemed more reasonable to knockdown SLA-II molecules by overexpressing the human dominant-negative CIITA gene (15). In summary, we believe that the knockout of B2M and CIITA had advantages and disadvantages: the advantages were that it prolonged the survival of xenografts and reduced the intensity of the immuno-suppressive regimen in xenotransplantation, and the disadvantages were that it harmed the health of pigs and increased the difficulty of breeding. Currently, although the CRISPR/Cas9 technology is simple and versatile for editing the pig genome, the goal of developing genetically modified donor pigs for xenotransplantation is to enable pigs to be used in the clinic. Therefore, to generate the tailored xenotransplantation donor pigs, we strongly suggest that we should carefully consider which genes are essential and non-essential for modification and formulate accurate gene-editing strategies for specific tissues or organs so that the CRISPR/Cas9 technology can more effectively serve the development of donor pigs.

## Conclusions

In conclusion, we successfully generated the triple-gene (GGTA1, B2M, and CIITA)-modified pigs by the CRISPR/Cas9 system and SCNT. These pigs presented the absence of the expression of α1,3-galactosyltransferase and SLA-II and the downregulation of the expression of SLA-I, which can enhance the ability of immunological tolerance in pig-to-human xenotransplantation. However, CIITA knockout caused a significant decrease in a cluster of SLA-II molecules, involved in immune- and infection-relevant pathways, including antigen processing and presentation and viral myocarditis, and made these pigs susceptible to pathogenic microorganisms.

## Data Availability Statement

The datasets presented in this study can be found in online repositories. The names of the repository/repositories and accession number(s) can be found at: https://ngdc.cncb.ac.cn/gsub/submit/gsa/subCRA008147/finishedOverview, CRA005606.

## Ethics Statement

The animal study was reviewed and approved by Animal Care and Use Committee of Yunnan Agricultural University (permission code: YAUACUC01). Written informed consent was obtained from the owners for the participation of their animals in this study.

## Author Contributions

H-JW, H-YZ, HY, and KX designed the research. SC, KX, HY, YZ, CY, JW, HL, and BJ performed the molecular experiments and analyzed data. DJ transfected the cell and performed a cell culture. JG, H-JW, HZ, TN, and TW carried out SCNT and embryo transfer. KX wrote the manuscript. H-YZ, XH, MAJ, and H-JW revised the manuscript and validated the data. All authors contributed to the article and approved the submitted version.

## Funding

The work was supported by the grants from the National Key R&D Program of China (grant no. 2019YFA0110700), the Major Science and Technology Project of Yunnan Province (grant no. 202102AA310047), and the Innovative Research Team of Science and Technology in Yunnan Province.

## Conflict of Interest

The authors declare that the research was conducted in the absence of any commercial or financial relationships that could be construed as a potential conflict of interest.

## Publisher's Note

All claims expressed in this article are solely those of the authors and do not necessarily represent those of their affiliated organizations, or those of the publisher, the editors and the reviewers. Any product that may be evaluated in this article, or claim that may be made by its manufacturer, is not guaranteed or endorsed by the publisher.

## Supplementary Material

Supplementary Figure 1 Verification of RNA-seq data quality. **(A)** The Pearson correlation coefficient between GBC-modified and WT pigs. **(B)** Boxplots of the gene expression distributions.

Supplementary Figure 2 Expression levels of GGTA1, B2M, and CIITA genes analyzed by RNA-seq in the PBMCs of the pig's spleen.

Supplementary Table 1 Sample information.

Supplementary Table 2 The primers of qPCR.
