# Supplementary figures and images for: Production of Triple-Gene (GGTA1, B2M and CIITA)-Modified Donor Pigs for Xenotransplantation
Source: Front Vet Sci. 2022 Apr 28;9:848833. doi: 10.3389/fvets.2022.848833 (PMC9097228; doi:10.3389/fvets.2022.848833)

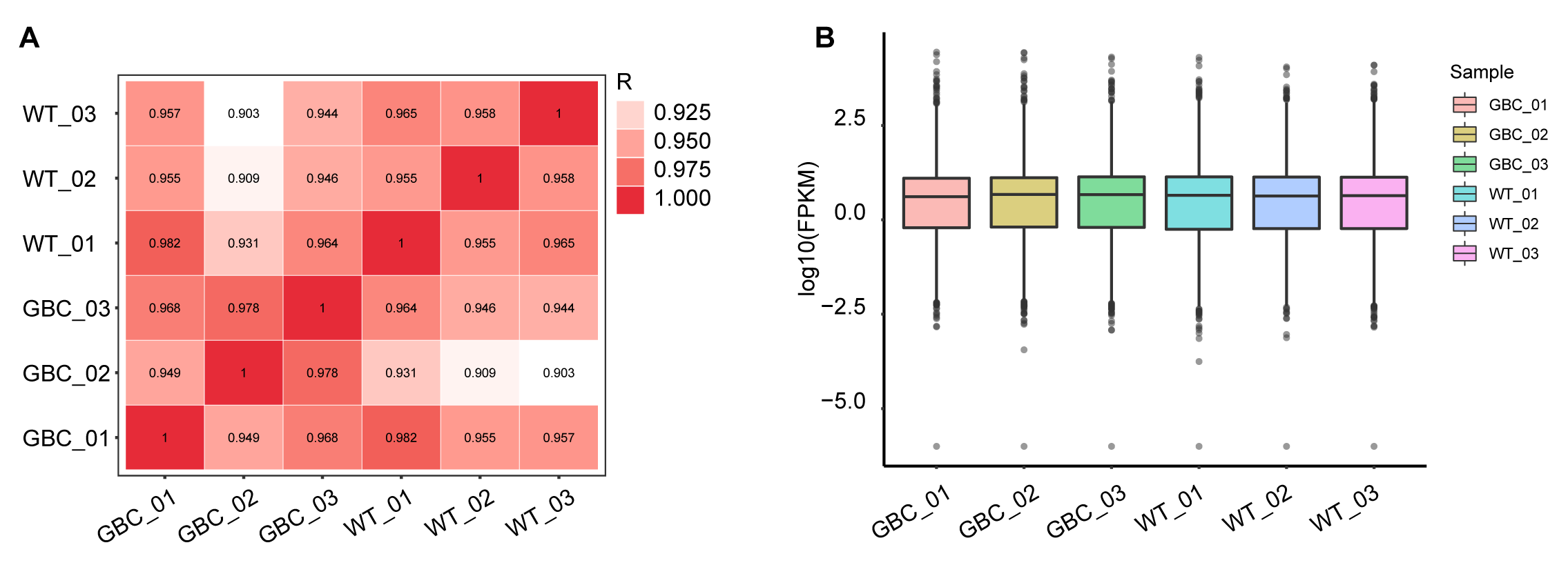

Supplement: Supplementary Figure 1 — Verification of RNA-seq data quality. (A) The Pearson correlation coefficient between GBC-modified and WT pigs. (B) Boxplots of the gene expression distributions. [file Image_1.tif]

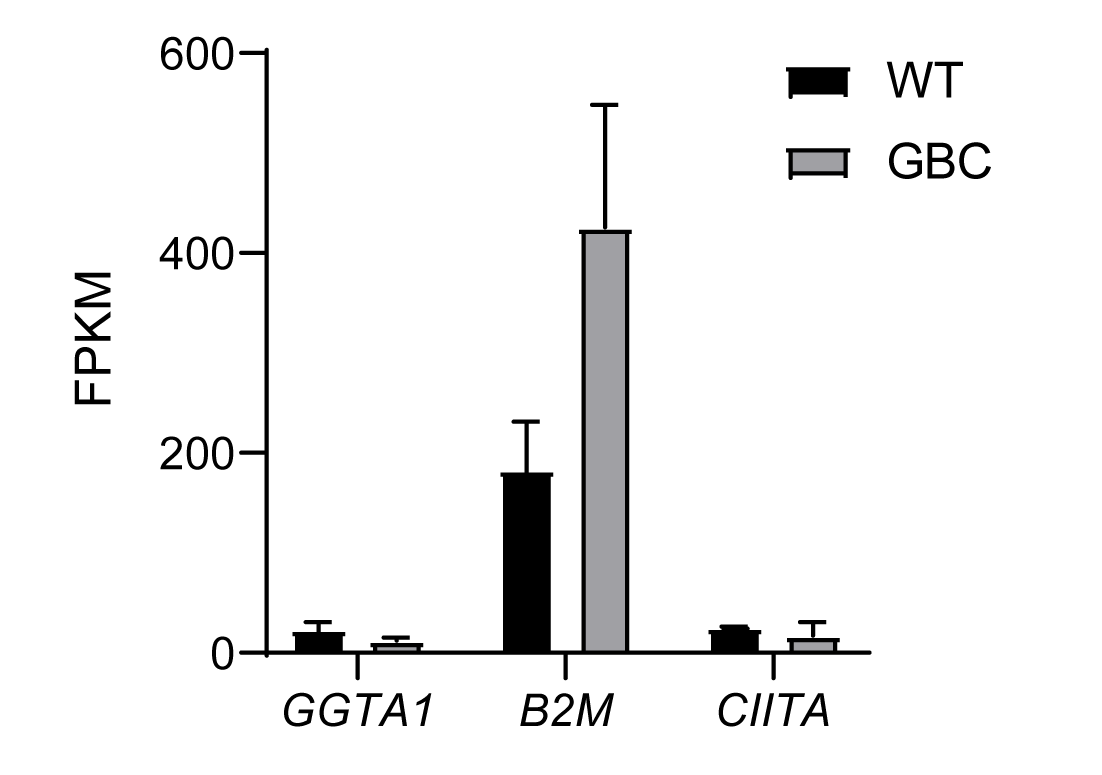

Supplement: Supplementary Figure 2 — Expression levels of GGTA1, B2M, and CIITA genes analyzed by RNA-seq in the PBMCs of the pig's spleen. [file Image_2.tif]
