# Supplementary material for: Production of Triple-Gene (GGTA1, B2M and CIITA)-Modified Donor Pigs for Xenotransplantation
Source: Front Vet Sci. 2022 Apr 28;9:848833. doi: 10.3389/fvets.2022.848833 (PMC9097228; doi:10.3389/fvets.2022.848833)
Supplement: Supplementary Table 1 — Sample information. [file Table_1.docx]

**Table S1 Sample information**

| **No.** | **Name** | **Genotype** | **Donor** | **Experiment** |
| --- | --- | --- | --- | --- |
| **1** | P1 | **GGTA1:**  GCTACAGGCCTGGTGGTtACAAGG (+1)  **B2M:**  CCCGAAG -TTCAGGTTTACTCAC (△1)  CCCGAAG - - - CAGGTTTACTCAC (△3)  CCCGAAGGTTCAGGTTTACTCAC (WT)  **CIITA:**  CCATCA-CTGCGAACAGTTCAGC (△1)  CCATCA - - - - - - - - - - GTTCAGC (△10) | C9 colony | q-PCR (GGTA1, B2M, CIITA) |
| **2** | P2 | **GGTA1:**  GCTACAGGCCTGGTGGTtACAAGG (+1)  GCTACAGGCCTGGTGGTACAAGG (WT)  **B2M:**  CCCGAAG -TTCAGGTTTACTCAC (△1)  CCCGAAG - - - CAGGTTTACTCAC (△3)  CCCGAAGGTTCAGGTTTACTCAC (WT)  **CIITA:**  CCATCAACTGCGAACAGTTCAGC (WT)  CCATCA - - - - - - - - - - GTTCAGC (△10) | C9 colony |  |
| **3** | P3 | **GGTA1:**  GCTACAGGCCTGGTGGTtACAAGG (+1)  **B2M:**  CCCGAAG -TTCAGGTTTACTCAC (△1)  CCCGAAG - - - CAGGTTTACTCAC (△3)  CCCGAAGGTTCAGGTTTACTCAC (WT)  **CIITA:**  CCATCA-CTGCGAACAGTTCAGC (△1)  CCATCA - - - - - - - - - - GTTCAGC (△10) | C9 colony | q-PCR (GGTA1, B2M, CIITA) |
| **4** | P4 | **GGTA1:**  GCTACAGGCCTGGTGGTtACAAGG (+1)  **B2M:**  CCCGAAG -TTCAGGTTTACTCAC (△1)  CCCGAAG - - - CAGGTTTACTCAC (△3)  CCCGAAGGTTCAGGTTTACTCAC (WT)  **CIITA:**  CCATCA-CTGCGAACAGTTCAGC (△1)  CCATCA - - - - - - - - - - GTTCAGC (△10) | C9 colony | q-PCR (GGTA1, B2M, CIITA, SLA-I, SLA-II), IHC |
| **5** | P5 | **GGTA1:**  GCTACAGGCCTGGTGGTtACAAGG (+1)  **B2M:**  CCCGAAG -TTCAGGTTTACTCAC (△1)  CCCGAAG - - - CAGGTTTACTCAC (△3)  CCCGAAGGTTCAGGTTTACTCAC (WT)  **CIITA:**  CCATCA-CTGCGAACAGTTCAGC (△1)  CCATCA - - - - - - - - - - GTTCAGC (△10) | C9 colony | q-PCR (GGTA1, B2M, CIITA) |
| **6** | P6 | **GGTA1:**  GCTACAGGCCTGGTGGTtACAAGG (+1)  **B2M:**  CCCGAAG -TTCAGGTTTACTCAC (△1)  CCCGAAG - - - CAGGTTTACTCAC (△3)  CCCGAAGGTTCAGGTTTACTCAC (WT)  **CIITA:**  CCATCA-CTGCGAACAGTTCAGC (△1)  CCATCA - - - - - - - - - - GTTCAGC (△10) | C9 colony |  |
| **7** | P4P1 | **GGTA1:**  GCTACAGGCCTGGTGGTtACAAGG (+1)  **B2M:**  CCCGAAG -TTCAGGTTTACTCAC (△1)  CCCGAAG - - - CAGGTTTACTCAC (△3)  CCCGAAGGTTCAGGTTTACTCAC (WT)  **CIITA:**  CCATCA-CTGCGAACAGTTCAGC (△1)  CCATCA - - - - - - - - - - GTTCAGC (△10) | P4 | Autopsy |
| **8** | P4P2 | **GGTA1:**  GCTACAGGCCTGGTGGTtACAAGG (+1)  **B2M:**  CCCGAAG -TTCAGGTTTACTCAC (△1)  CCCGAAG - - - CAGGTTTACTCAC (△3)  CCCGAAGGTTCAGGTTTACTCAC (WT)  **CIITA:**  CCATCA-CTGCGAACAGTTCAGC (△1)  CCATCA - - - - - - - - - - GTTCAGC (△10) | P4 | Autopsy |
| **9** | P4P3 | **GGTA1:**  GCTACAGGCCTGGTGGTtACAAGG (+1)  **B2M:**  CCCGAAG -TTCAGGTTTACTCAC (△1)  CCCGAAG - - - CAGGTTTACTCAC (△3)  CCCGAAGGTTCAGGTTTACTCAC (WT)  **CIITA:**  CCATCA-CTGCGAACAGTTCAGC (△1)  CCATCA - - - - - - - - - - GTTCAGC (△10) | P4 | IF (B2M, CIITA, SLA-I, SLA-II DR), FC (SLA-I, SLA-II DR, SLA-DQ, Antibody reaction, MLR) |
| **10** | GBC_1 | **GGTA1:**  GCTACAGGCCTGGTGGTtACAAGG (+1)  **B2M:**  CCCGAAG -TTCAGGTTTACTCAC (△1)  CCCGAAG - - - CAGGTTTACTCAC (△3)  CCCGAAGGTTCAGGTTTACTCAC (WT)  **CIITA:**  CCATCA-CTGCGAACAGTTCAGC (△1)  CCATCA - - - - - - - - - - GTTCAGC (△10) | P4P1 | RNA-seq |
| **11** | GBC_2 | **GGTA1:**  GCTACAGGCCTGGTGGTtACAAGG (+1)  **B2M:**  CCCGAAG -TTCAGGTTTACTCAC (△1)  CCCGAAG - - - CAGGTTTACTCAC (△3)  CCCGAAGGTTCAGGTTTACTCAC (WT)  **CIITA:**  CCATCA-CTGCGAACAGTTCAGC (△1)  CCATCA - - - - - - - - - - GTTCAGC (△10) | P4P1 | RNA-seq |
| **12** | GBC_3 | **GGTA1:**  GCTACAGGCCTGGTGGTtACAAGG (+1)  **B2M:**  CCCGAAG -TTCAGGTTTACTCAC (△1)  CCCGAAG - - - CAGGTTTACTCAC (△3)  CCCGAAGGTTCAGGTTTACTCAC (WT)  **CIITA:**  CCATCA-CTGCGAACAGTTCAGC (△1)  CCATCA - - - - - - - - - - GTTCAGC (△10) | P4P1 | RNA-seq |
| **13** | GBC_14 | **GGTA1:**  GCTACAGGCCTGGTGGTtACAAGG (+1)  **B2M:**  CCCGAAG -TTCAGGTTTACTCAC (△1)  CCCGAAG - - - CAGGTTTACTCAC (△3)  CCCGAAGGTTCAGGTTTACTCAC (WT)  **CIITA:**  CCATCA-CTGCGAACAGTTCAGC (△1)  CCATCA - - - - - - - - - - GTTCAGC (△10) | P4P1 | Analysis of T cell subtype by FC |
