# Supplementary material for: Production of Triple-Gene (GGTA1, B2M and CIITA)-Modified Donor Pigs for Xenotransplantation
Source: Front Vet Sci. 2022 Apr 28;9:848833. doi: 10.3389/fvets.2022.848833 (PMC9097228; doi:10.3389/fvets.2022.848833)
Supplement: Supplementary Table 2 — The primers of qPCR. [file Table_2.docx]

Table S2. The primers of q-PCR

| Gene | Primers sequence (5’ to 3’) | |
| --- | --- | --- |
| GGTA1 | F: ACAAGGCACATCCTGACGAGTTC | R: CCTTGAAGCACTCCTGAGTGATG |
| B2M | F: TTCTACCTTCTGGTCCACACTG | R: TCTTGGGCTTATCGAGAGTCAC |
| CIITA | F: CCAGGCACTGGAGGAGAAATTTAC | R: TCCAGATGCTGCAGGGAAGAAAAG |
| SLA-I | F: CCTCTTCCTGCTGCTGTCG | R: ACTCCACACACAGTCCCTGC |
| SLA-II DOB | F: ATGACTCAAGGCAGAGATTC | R: TCAACAAGGCAGGTATAGAC |
| SLA-II DQB1 | F: GGCGAGTGCTACTTCTAC | R: GTCCAGTCTCCGTTCCTA |
| SLA-II DRA | F: ACCAGAGGAGTGTCAGAG | R: CCAGTGCTTGAGAAGAGG |
| SLA-II DRB1 | F: GCAGACACAACTACAGGAT | R: GAAGGTCCAGTCTCCATTAG |
| GAPDH | F: TACCCCTCCTCTGATGTCCTGAG | R: TCGTGGAGGGACTCATGGTAGG |
